# Anti-inflammatory therapies are associated with delayed onset of anemia and reduction in transfusion requirements in critically ill patients: results from two studies

**DOI:** 10.1186/s13054-024-04898-z

**Published:** 2024-04-09

**Authors:** Madelief Bolscher, Stephanie C. E. Koster, Matty Koopmans, Jelle L. G. Haitsma Mulier, Lennie P. G. Derde, Nicole P. Juffermans

**Affiliations:** 1 Department of Intensive Care, OLVG Hospital, Oosterpark 9, Amsterdam, The Netherlands; 2 Department of Intensive Care, Zaans Medisch Centrum, Zaandam, The Netherlands; 3 Department of Intensive Care, University Medical Center Utrecht, Utrecht, The Netherlands; 4 Department of Intensive Care, Erasmus Medical Center, Molewaterplein 40, Rotterdam, The Netherlands; 5 Laboratory of Translational Intensive Care, Erasmus Medical Center, Molewaterplein 40, Rotterdam, The Netherlands

**Keywords:** COVID-19, Anemia, Transfusion, Anti-inflammatory treatment, Steroids

## Abstract

**Background:**

Anemia is a hallmark of critical illness, which is largely inflammatory driven. We hypothesized that the use of anti-inflammatory agents limits the development of anemia and reduces the need for red blood cell (RBC) transfusions in patients with a hyper-inflammatory condition due to COVID-19.

**Methods:**

An observational cohort (*n* = 772) and a validation cohort (a subset of REMAP-CAP, *n* = 119) of critically ill patients with hypoxemic respiratory failure due to COVID-19 were analyzed, who either received no treatment, received steroids or received steroids plus IL-6 blocking agents. The trajectory of hemoglobin (Hb) decline and the need for RBC transfusions were compared using descriptive statistics as well as multivariate modeling.

**Results:**

In both cohorts, Hb level was higher in the treated groups compared to the untreated group at all time points. In the observational cohort, incidence and number of transfused patients were lower in the group receiving the combination treatment compared to the untreated groups. In a multivariate analysis controlling for baseline Hb imbalance and mechanical ventilation, receipt of steroids remained associated with a slower decline in Hb level and the combination treatment remained associated with a slower decline of Hb and with less transfusions. Results remained the same in the validation cohort.

**Conclusion:**

Immunomodulatory treatment was associated with a slower decline in Hb level in critically ill patients with COVID-19 and with less transfusion. Findings point toward inflammation as an important cause for the occurrence of anemia in the critically ill.

**Supplementary Information:**

The online version contains supplementary material available at 10.1186/s13054-024-04898-z.

## Introduction

During their stay in the intensive care unit (ICU), the majority of critically ill patients develops anemia. 40–60% of patients have a low hemoglobin (Hb) level already at ICU admission, and > 80% of the patients is anemic after 1 week [1, 2]. Anemia persists during ICU stay and even up to 12 months after ICU discharge in half of the anemic patients [3]. Anemia hampers oxygen delivery capacity. Thereby, if compensatory mechanisms of the cardio-respiratory system fall short, anemia can contribute to organ failure in general as well as increase the risk of ischemic events. Collectively, anemia is associated with increased short- and long-term mortality [4]. In addition, anemia hampers rehabilitation after ICU discharge. Therefore, anemia is a clinically relevant problem in the ICU, as well as in the convalescent phase of critical illness.

Although the pathogenesis of anemia in the critically ill may be multifactorial, including dietary deficiencies and frequent phlebotomy, anemia of inflammation is thought to be the most important cause of anemia [5, 6]. Increased concentrations of cytokines, in particular IL-6, induce the synthesis of hepcidin, which inhibits iron availability for erythropoiesis [7]. In addition, cytokines activate macrophages, with subsequent phagocytosis of erythrocytes and hence a decrease in the lifespan of erythrocytes [8]. Consequently to a low bio-availability, iron supplementation has limited efficacy in critically ill patients with anemia of inflammation [9]. Thereby, anemia is often treated with red blood cell (RBC) transfusion. Despite a widely adopted restrictive policy, a considerable proportion of 30–50% of ICU patients still receives RBC transfusion [10, 11], rendering this an important therapeutic intervention. However, there may be an association between RBC transfusion and adverse outcome, including long-term mortality [12].

Since both anemia and RBC transfusion are associated with adverse outcome, prevention of anemia may beneficially impact outcome. As inflammation drives the development of anemia, we hypothesize that anti-inflammatory treatments may slow this process. In line with this thought, receipt of IL-6 receptor blockers reduces the development of anemia in patients with rheumatoid arthritis [13]. The impact of anti-inflammatory agents on anemia in the critically ill is, however, not known.

The Coronavirus disease 2019 (COVID-19) pandemic resulted in a cohort of critically ill patients with a homogenous severe inflammatory condition. During the waves of the pandemic, treatment with steroids [14–16] and IL-6 blocking agents [17] was found to be effective and introduced in clinical care. This study investigated the impact of these immunomodulating agents on the occurrence of anemia in two cohorts of critically ill COVID-19 patients.

## Methods

### Observational patient cohort

All patients > 18 years of age admitted to the ICU of 2 hospitals in The Netherlands (OLVG teaching hospital in Amsterdam, or Zaans Medisch Centrum in Zaandam) because of hypoxemic respiratory failure due to COVID-19 (PCR proven) between March 2020 and March 2022 were included. Exclusion criteria were: receipt of immunosuppressive medication prior to hospital admission (systemic corticosteroids, methotrexate, mycophenolate, tacrolimus, chemotherapy, biologicals), pre-existing kidney disease with eGFR < 60 ml/min, confirmed pregnancy, malignant neoplasm, ICU stay < 12 h, liver disease, angiodysplasia, disease of iron metabolism, or bleeding requiring RBC transfusion. Treatment of COVID-19 was largely with supportive care, with oxygen support via high-flow nasal cannula or invasive mechanical ventilation, with proning in the most severely hypoxemic patients. No patients received treatment with extra corporeal membrane oxygenation. In July 2020, prescription of dexamethasone (6–12 mg daily) [14, 15] was implemented in the treatment protocol of both hospitals, with addition of tocilizumab (400 mg once) in December 2020. The study population was divided in the following three groups based on the received treatment during ICU stay: no treatment; only steroids; steroids and IL-6 inhibitors. The local IRB waived the need for informed consent for this retrospective study (WO20.108).

### Validation cohort

Given that observational data can be subjected to bias, an additional analysis was done in another cohort of patients with data that was accessible to the investigators as part of the REMAP-CAP trial. Patients with respiratory failure due to COVID-19 (PCR positive or CT scan with CO-RADS 4 or more) admitted to the ICU of the UMCU and who were randomized into any domain of the REMAP-CAP trial between March 2020 and February 2022 were included in this sub-analysis. As recruitment of these patients differed from those in the observational cohort study, data from the two studies were not lumped together but analyzed separately. Unfortunately, of the patients included in the REMAP-CAP, Hb levels and transfusion data were only available for patients included at the University Medical Center Utrecht (UMCU), and hence, only those were included in this sub-analysis. Patients were excluded if the duration of their ICU admission was < 5 days or if no informed consent for the REMAP-CAP study was obtained from the patient or their legal representative. The study groups were tocilizumab, sarilumab, corticosteroids only, or no immune modulation. Patients receiving tocilizumab or sarilumab were always simultaneously treated with corticosteroids. In the analysis, patients treated with either sarilumab or tocilizumab were pooled, as the effect size and mechanism of action for both these IL-6 inhibitors are highly comparable.

### Definitions

Anemia was defined according to the definition of the World Health Organization (WHO) as blood hemoglobin values of < 13g/dl in men and < 12g/dl in women. ICU admission day was defined as day 1. Bleeding was identified with chart review, in which bleeding was scored as ‘yes’ (and patients hence excluded from the analysis) if bleeding was mentioned in the list of problems or in the discharge letter, in combination with receipt of RBC transfusion.

### Transfusion policy

According to transfusion protocols at all hospitals, RBC transfusion was indicated at a trigger level of 7 g/dl.

### Data and outcomes

Baseline data included demographics, co-morbidity, Acute Physiology and Chronic Health Evaluation (APACHE) IV score, Sequential Organ Failure Assessment score (SOFA) score, medications, and daily laboratory values. Data were extracted from electronic health records for all patients. The a priori chosen primary outcome of this study was declined in Hb level between days 1 and 7. This time span of 7 days was taken because of loss of power due to ICU discharge of patients beyond this time frame. Secondary outcomes included the need for RBC transfusion, number of RBC transfusions (units) per patient, duration between admission, receipt of the first RBC transfusion (days), and markers of hemolysis. Other data that were gathered included the use of invasive mechanical ventilation, duration of invasive mechanical ventilation, need for renal replacement therapy, development of AKI during ICU stay (defined as doubling of creatinine or > 50% decrease in glomerular filtration rate), occurrence of bacterial superinfection (defined as a clinical suspicion for infection in combination with a positive culture from a relevant specimen), invasive pulmonary aspergillosis (probable, according to [18]), and occurrence of death while on ICU.

### Statistical analysis

Analysis was performed using Statistical Package for Social Sciences. Continuous variables are expressed as mean/standard deviation or median/interquartile range, depending on their distribution. Categorical variables are expressed as total number and percentage. All data were checked for normal distribution, using the Kolmogorov–Smirnov Normal test. We compared means or medians between the three groups using Kruskal–Wallis with Bonferroni correction. Also, in the observational cohort, multiple linear regression analysis was done for continuous outcome measures with adjustment for the stratification variable (no treatment, steroids or steroids and IL-6 inhibitors) and statistically significant differences at baseline (hypertension and baseline Hb level). In a second model, after checking for collinearity, CRP and duration of mechanical ventilation were also added as covariates. The outcome of the models was the difference between Hb level between day 1 and day 7, amount of transfusion, and proportion of transfused patients.

## Results

### Observational cohort

Of 772 patients assessed for eligibility, 3 patients were excluded because of immunosuppressive medication, 21 patients were excluded because ICU stay was < 12 h due to transferal because of limited bed availability, 29 patients were excluded because COVID was not confirmed by PCR, and 37 patients were excluded because of bleeding with a need for RBC transfusion, leaving 682 patients included in this analysis.

Patients had a median age of 64 [56–72] years and were predominantly male (72.1%), with a median BMI of 29 kg/m^2^. Except for the presence of hypertension, that was statistically significantly less prevalent in the non-treated group, characteristics and disease severity did not differ between the 3 cohorts (Table 1). There was an imbalance in laboratory values on ICU admission, with statistically significant higher Hb values and lower CRP values in the treated groups compared to the untreated group. The amount of patients on mechanical ventilation and/or on vasopressors within 48 h was similar. In line, SOFA scores on ICU admission day were also similar.

**Table 1:** Characteristics of COVID-19 patients in observational cohort

	Total (*N* = 682)	No treatment (*N* = 155)	Steroids (*N* = 324)	Steroids and IL-6 inhibitors (*N* = 201)	*P*-value
Age (yrs)	64 [56–72]	65 [56–73]	64 [56–72]	62 [55–72]	0.57
Male, *n* (%)	492 (72.1)	117 (75.5)	242 (74.7)	133 (66.2)	0.06
BMI	29 [26–33]	29 [26–32]	29 [26–33]	29 [26–33]	0.29
*Co-morbidity*					
DM *n*, (%)	207 (30.4)	52 (33.5)	104 (32.1)	50 (24.9)	0.13
Hypertension *n*, (%)	245 (35.9)	44 (28.4)	132 (40.7)	68 (33.8)	0.02
Heart failure *n*, (%)	24 (3.5)	10 (6.5)	8 (2.5)	6 (3.0)	0.08
CAD n, (%)	101 (14.8)	27 (17.4)	46 (14.2)	27 (13.5)	0.55
*Medication use on admission*					
PLT inhibitors *n*, (%)	127 (18.6)	28 (18.1)	61 (18.8)	38 (19.0)	0.97
Anticoagulants *n*, (%)	216 (31.7)	41 (26.5)	110 (34.0)	65 (32.5)	0.25
*Disease severity*					
APACHE III	61 [49–75]	60 [47–77]	62 [51–76]	60 [49–74]	0.48
SOFA	6 [4–8]	6 [4–7]	6 [4–8]	7 [4–8]	0.82
IMV *n*, (%)	513 (75.2)	113 (72.9)	251 (77.5)	148 (73.6)	0.45
Vasopressor > 2 days *n*, (%)	341 (50.0)	79 (51.0)	158 (48.8)	104 (51.7)	0.80
*Lab on ICU admission*					
Hb (g/dl)	12.1 [10.8–13.2]	11.4 [10.2–12.6]	12.2 [10.8–13.2]*	12.6 [11.4–13.5]*	< 0.001
CRP (mg/L)	104 [52–179]	167 [88–220]	91 [43–162]*	84 [57–164]*	< 0.001
LDH (U/L)	409[324–512]	395[300–508]	405[324–501]	430[342–532]	0.046

*P < 0.05 vs no treatment

*BMI* body mass index, *DM* diabetes mellitus, *CAD* coronary artery disease, *PLT* platelet, *APACHE* Acute Physiology and Chronic Health Evaluation, *SOFA* Sequential Organ Failure Assessment, *LDH* lactate dehydrogenase

### Association of anti-inflammatory treatments on Hb trajectory and transfusion requirements in the observational cohort

**Fig. 1:**
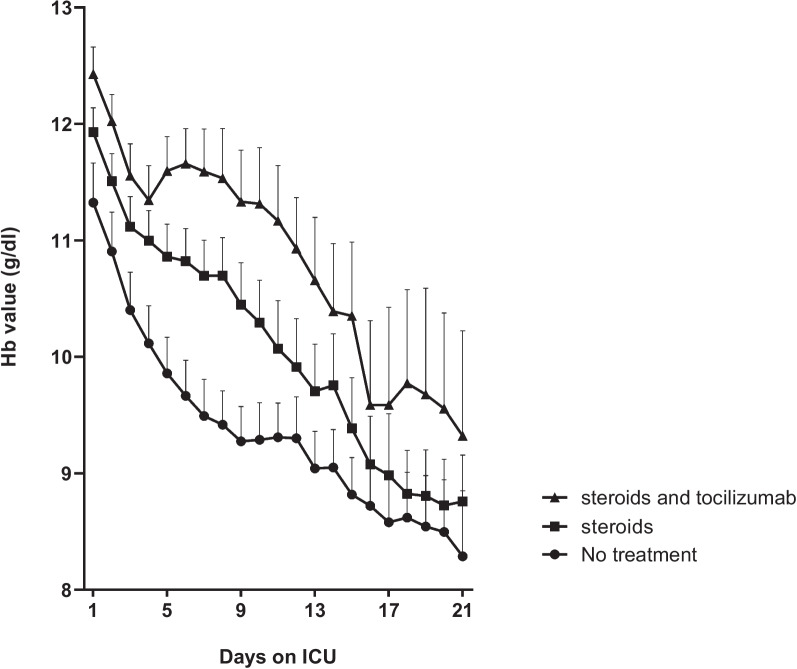
Hb trajectory in an observational cohort of COVID-19 patients

The occurrence of anemia during ICU stay was high in all groups. Anti-inflammatory treatment was associated with a significant reduction in the proportion of patients developing anemia, which occurred in 75% of patients receiving steroids, in 70% of patients receiving combination treatment and in 87% of untreated patients. The trajectory of the Hb level throughout the observation period of 21 days is depicted in Fig. 1. At all time points, the group not receiving anti-inflammatory agents had the lowest Hb level of all groups. The median decline in Hb level between ICU admission and day 7 was − 1.8 [− 2.6 to − 0.8] g/dl in untreated patients, which was significantly greater compared to those treated with steroids (− 1.3 [− 2.3 to − 0.2] g/dl) and compared to those treated with a combination of steroids and IL-6 inhibition (− 0.5[− 1.6 to 0.3] g/dl, *P* < 0.001 for group differences).

The proportion of patients receiving RBC transfusion during the 21-day observation period on ICU was lower both in patients receiving steroids and in patients receiving combination treatment when compared to the non-treated group (Table 2). Also, in the patients receiving combination treatment, but not in those only receiving steroids, the number of RBC transfusions per patient was lower and the time elapsed from ICU admission until the first transfusion was longer compared to untreated patients. During ICU stay, changes in LDH and bilirubin values did not differ over time (data not shown).

**Table 2:** Anemia and transfusion outcomes of COVID-19 patients in observational cohort

	Total (*N* = 682)	No treatment (*N* = 155)	Steroids (*N* = 324)	Steroids and IL-6 inhibitors (*N* = 201)	*P*-value
Developing anemia *n*, (%)	517 (76%)	133 (86%)	243 (75)	140 (70%)**	< 0.001
Patients transfused *n*, (%)	104 (15%)	37 (24%)	48 (15%)*	19 (10%)**	< 0.001
No. of RBC units/patient	2 [1–5]	3 [2–8]	3 [1–5]	1 [0–2]**	< 0.001
Days until 1st transfusion	9 [3–17]	10 [4–17]	6 [3–14]	19 [4–25]*	0.03

*RBC* red blood cell

*P < 0.05 vs no treatment, **P < 0.01 vs no treatment,

### Association between treatments and co-morbidity during the course of disease in the observational cohort

Anti-inflammatory treatments were associated with less bacterial superinfection and a shorter duration of mechanical ventilation of 3 days in treated groups compared to the untreated group (Additional file 1: Table S1). This did not translate in clear differences in SOFA scores between groups over the course of ICU stay (Additional file 1: Fig. S1). As expected, the trajectory of CRP differed between treatment groups, showing an initial (further) increase in the untreated patients, whereas steroids and tocilizumab treatment was associated with an initial decline in CRP after ICU admission, rising again after 2–3 weeks (Additional file 1: Fig. S1).

### Multivariate modeling in observational cohort

Linear regression analyses were performed for the outcome Hb decline between day 1 and day 7 (in *n* = 323 patients for whom day 7 data were available) and for the outcome RBC transfusions (in the whole cohort). Baseline imbalances between groups were entered into the model, including admission Hb, CRP and hypertension. Also, given that the duration of mechanical ventilation was on average 3 days shorter in the treated groups compared to the untreated groups and that mechanical ventilation can contribute to the development of anemia [19], duration of mechanical ventilation was also entered into the models. The variables that did not impact outcome were left out of the specific model. For the outcome of Hb decline, admission of CRP, admission of Hb and duration of MV significantly decreased Hb. When adjusted for these variables, steroids as mono-treatment were associated with an increase in Hb level between day 1 and day 7 (*p* = 0.039, Additional file 1: Table S3). Also, the combination treatment was associated with an increase in Hb level, with a slightly stronger effect size (Additional file 1: Table S4). For the outcome of RBC transfusions, only MV duration significantly decreased the number of RBC transfusions. When corrected for MV duration, steroids as monotherapy did not impact the number of RBC transfusions (Additional file 1: Table S5), but the combination therapy remained associated with a lower amount of RBC transfusions per patient, with an average of 1.9 units (Additional file 1: Table S6). In addition, regarding the time until first RBC transfusion, the combination treatment was also an independent factor, with a much stronger impact than mechanical ventilation (Beta 7.49 vs Beta 0.12).

### Findings in validation cohort

**Fig. 2:**
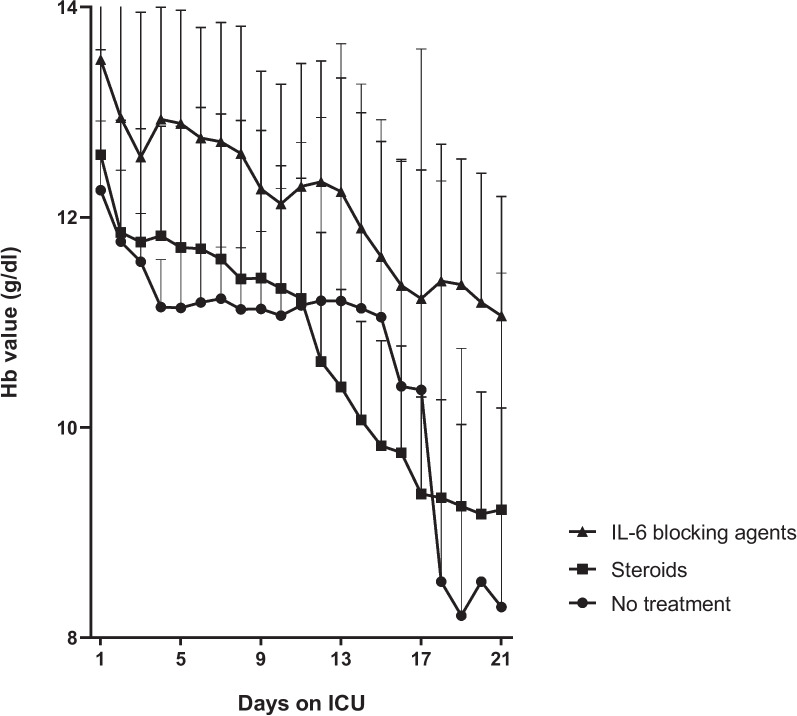
Hb trajectory in a validation (randomized) cohort of COVID-19 patients

Given baseline imbalances in the observational cohort, with less sick patients in the treated groups, which could have influenced the slower onset of anemia independent of treatment, an additional analysis was done in a sub-study of the REMAP-CAP trial in patients admitted to a single center. A total of 119 patients were included, of which *n* = 11 received no treatment, *n* = 34 received steroids and *n* = 74 received IL-6 blockers. Demographics were similar to the observational cohort (Additional file 1: Table S2). Again, a lower admission of CRP and a higher admission of Hb level were noted in the treated groups compared to the untreated groups, but SOFA score did not differ between groups. The decline in Hb showed a similar trend as in the observational cohort (Fig. 2). Due to lower numbers, additional analyses such as multivariate modeling were not deemed useful.

## Discussion

The incidence of anemia in these cohorts of COVID-related hypoxemia following ICU admission is high when compared to other critically ill patient cohorts [3]. Furthermore, this study indicates that treatment with steroids and IL-6 inhibitors is associated with delayed onset of anemia, with a subsequent decrease both in the proportion of transfused patients and in the amount of transfusion per patient. These findings are in line with those in patients with other inflammatory conditions, such as rheumatoid arthritis [20]. Collectively, findings suggest inflammation as a causative factor in the occurrence of anemia.

Patients treated with a combination of steroids and IL-6 inhibitors had the slowest decline in Hb levels and less often needed RBC transfusion compared to patients treated with steroids alone. This effect supports the important role of IL-6 in the pathogenesis of anemia of inflammation. IL-6 hampers the proliferation of erythroid precursors and decreases levels of erythropoietin and iron [21]. Also, IL-6 increases production of hepcidin, which in turn limits the uptake of iron from the intestines and causes macrophage iron sequestration [8, 22].

In these analyses, the impact of steroids as monotherapy on RBC transfusion is less clear. An effect of steroids on the onset of anemia was seen, but steroids did not significantly reduce the number of RBC transfusions when corrected for other factors. Corticosteroids were shown to inhibit erythrophagocytosis in vitro [23], but whether steroids are helpful in patients with anemia due to chronic inflammation, such as in rheumatoid arthritis, is less clear [24]. Possibly, the effects of anti-inflammatory agents on anemia may be ‘dose-dependent’, implying that more intensified treatment with anti-inflammatory agents is associated with a slower decline in Hb levels and a greater decrease in transfusion requirements.

There was a baseline imbalance in Hb levels in the observational cohort and to a lesser extend also in the validation cohort, with higher Hb levels on admission in the treated groups. We do not think that this is by chance. As patients in the treatment groups also clearly had lower CRP levels on admission, and the treatment protocol in both hospitals stated that hypoxemic patients should receive treatment regardless of whether they were on ER, ward or ICU, we postulate that therapy was administered to hypoxic patients already early after hospital admission, contributing to this imbalance already at ICU admission. Also, when we corrected for lower admission Hb and CRP with statistical modeling, an impact remained on the number of RBC transfusions.

An alternative explanation for the delayed onset of anemia may be that patients needed less intensified treatment in general, in particular mechanical ventilation. Indeed, the duration of mechanical ventilation was an important factor in the occurrence of anemia. However, when using a model adjusted for duration of mechanical ventilation, immunomodulatory treatment remained significantly associated with slower onset of anemia and less transfusions. Another explanation is that fluid balance may have affected the Hb level. Unfortunately, we do not have data on fluid balance.

This study has some important limitations. As noted, there were baseline imbalances between groups. Second, over time, not only pharmacologic treatment changed, but possibly also other management strategies such as settings of mechanical ventilation. We did not control for all variables that can potentially influence the Hb trajectory in the model. However, similar trends were observed in the two different cohorts. Also, viral strains probably changed. However, while the proportion of patients developing severe illness due to COVID-19 reduced over time and with other dominant viral strains, once patients develop severe disease needing ICU admission, the clinical symptoms of COVID-related hypoxemia remained the same in all waves of the pandemic. In line with this, SOFA scores on admission did not differ between groups. Finally, the observation period was 21 days of ICU admission and the multivariate analysis on Hb value was limited to 7 days, due to a loss of power as patients were dismissed from ICU. Therefore, we do not know longer-term effects. However, as anemia does not improve during ICU stay due to inflammatory-driven inhibition of erythropoiesis, we presume that a longer observation period would have yielded an even bigger difference in RBC transfusion rate between treated and untreated groups. Also, we did not verify transfusion policy. The last limitation is that following ICU admission, a proportion of patients was lost to follow-up due to transport between hospitals, because of shortage of ICU beds, resulting in fewer data points over time.

Results may, however, have implications. The prevention of anemia in ICU patients by targeting inflammation is a novel angle. Obviously, chronic anemia such as in rheumatoid arthritis is different than the acute hyper-inflammatory reactions as often seen in the critically ill, including those with hypoxemic COVID-19. Thereby, it may appear ‘farfetched’ to compare these syndromes. However, the pathophysiology of anemia of inflammation has large overlap within these different syndromes and IL-6 inhibitors have similar results on slowing onset of anemia. Nevertheless, results of this study should be regarded as hypothesis-generating only, given that confounding variables were not controlled for. Therefore, whether the risk–benefit profile of the use of anti-inflammatory agents as a novel therapy to prevent anemia is beneficial for critically ill patient populations with hyper-inflammatory syndromes such as COVID-19 remains to be determined.

In conclusion, in patients with COVID-related hypoxemia, anti-inflammatory agents are associated with delayed onset of anemia, with a subsequent decrease in transfusion requirements. Findings corroborate with inflammation being an important cause of anemia.

### Supplementary Information

**Additional file 1**. Supplementary tables and figures.

## Data Availability

All data generated or analyzed during this study are included in this published article.

## Abbreviations

APACHE: Acute Physiology and Chronic Health Evaluation
Hb: Hemoglobin
COVID-19: CoronaVirus Disease 2019
IL: Interleukin
PCR: Polymerase chain reaction
REMAP-CAP: Randomized embedded multifactorial adaptive platform for Community-acquired pneumonia
SOFA: Sequential Organ Failure Assessment score
