# Supplementary material for: Anti-inflammatory therapies are associated with delayed onset of anemia and reduction in transfusion requirements in critically ill patients: results from two studies
Source: Crit Care. 2024 Apr 9;28:114. doi: 10.1186/s13054-024-04898-z (PMC11003051; doi:10.1186/s13054-024-04898-z)
Supplement: Supplementary file 1 — Additional file 1. Supplementary tables and figures. [file 13054_2024_4898_MOESM1_ESM.docx]

Supplementary information revision

Supplementary Table 1. Co-morbidity and outcomes of COVID-19 patients in observational cohort

|  | Total  (N= 682) | | | No treatment (N=155) | Steroids (N=324) | Steroids and IL-6 inhibitors (N= 201) | P-value |
| --- | --- | --- | --- | --- | --- | --- | --- |
| Bacterial infection n,(%) | | 107 (16%) | | 40 (26%) | 45 (14%)** | 22 (11%)** | <0.001 |
| Fungal infection  n,(%) | | | 92 (14%) | 19 (12%) | 50 (15%) | 23 (11%) | 0.374 |
| Need for RRT  n,(%) | | | 54 (8%) | 14 (9%) | 29 (9%) | 11 (6%) | 0.304 |
| AKI during ICU  n,(%) | | | 371 (54%) | 101 (65%) | 179 (55%)** | 89 (44%)** | <0.001 |
| Duration MV (days) | | | 4.5 [1.4-11] | 7.3 [1.2-17] | 4.2 [1.4-10]* | 4.0 [1.7-8.6]* | 0.029 |
| Died on ICU n,(%) | | | 84 (12%) | 20 (13%) | 50 (15%) | 15 (7%)* | 0.015 |

AKI= acute kidney injury, RRT=renal replacement therapy, MV=invasive mechanical ventilation, ICU=Intensive Care Unit. *P<0.05 vs no treatment, **P<0.01 vs no treatment

Supplementary Table 2. Characteristics of COVID-19 patients in validation cohort

|  | Total  (n=119) | No treatment  (n=11) | Steroids  (n=34) | IL-6-inhibitors  (n=74) |
| --- | --- | --- | --- | --- |
| Age (yrs) | 65 [57-72] | 71 [60-72] | 66 [59-73] | 64 [56-70] |
| Male n, (%) | 74 (62) | 9 (82) | 20 (59) | 45 (61) |
| BMI | 29 [25-33] | 26 [24-29] | 28 [25-34] | 30 [26-34] |
| **Co-morbidity** |  |  |  |  |
| DM n, (%) | 24 (20.2) | 1 (9.1) | 7 (20.6) | 16 (21.6) |
| Hypertension  n, (%) | 45 (37.8) | 3 (27.3) | 12 (35.3) | 30 (40.5) |
| Heart failure n, (%) | 1 (0.8) | 0 (0) | 0 (0) | 1 (1.4) |
| CAD n, (%) | 3 (2.6) | 0 (0) | 2 (5.9) | 1 (1.4) |
| **Medication use on admission** | |  |  |  |
| Anti-PLT n, (%) | 33 (28) | 3 (27) | 10 (29) | 20 (27) |
| Anticoagulants n, (%) | 12 (10.1) | 1 (9.1) | 4 (11.8) | 7 (9.5) |
| **Disease severity** |  |  |  |  |
| SOFA-score | 6 [5-7] | 7 [6-8] | 6 [5-8] | 6 [5-7] |
| Vasoactive medication in first 24 hrs n, (%) | 85 (71) | 10 (91) | 28 (82) | 47 (64) |
| Invasive mechanical ventilation n, (%) | 94 (79) | 10 (91) | 29 (85) | 55 (74) |
| **Lab on ICU admission** |  |  |  |  |
| Hb (g/dl) | 12.8 (1.1) | 12.3 (1.0) | 12.6 (1.0) | 13.5 (1.1) |
| CRP (mg/L) | 98 [53-171] | 211 [179-270] | 93 [50-187]* | 90 [53-138]* |
| LDH (U/L) | 66 [57-83] | 68 [58-86] | 69 [61-83] | 63 [53-82] |

BMI = body mass index, DM = diabetes mellitus, CAD= coronary artery disease, PLT=platelet, SOFA= sequential organ failure assessment, CRP=C-reactive protein, LDH=lactate dehydrogenase. *P<0.05 vs no treatment

Supplementary Table 3. Impact of steroids adjusted for significant covariates on Hb trajectory between day 1 – 7

| Co-variate | Hb change (g/dl) | Adjusted for | P value |
| --- | --- | --- | --- |
| Per 1 point increase in admission Hb (g/dl) | Decrease 0.483 | MV duration and steroids | P<0.001 |
| Per 1 hour MV | Decrease 0.001 | Admission Hb and steroids | P<0.001 |
| Steroids y/n | Increase 0.298 | Admission Hb and MV duration | P=0.039 |

Supplementary Table 4. Impact of combination treatment adjusted for significant covariates on Hb trajectory between day 1-7

| Co-variate | Hb change (g/dl) | Adjusted for | P value |
| --- | --- | --- | --- |
| Per 1 point increase in admission Hb (g/dl) | Decrease 0.630 | MV duration, CRP and treatments | P<0.001 |
| Per 1 point increase in admission CRP (mg/L) | Decrease 0.003 | Admission Hb, MV duration and treatments | P=0.01 |
| Per 1 hour MV | Decrease 0.001 | Admission Hb, CRP and treatments | P=0.006 |
| Steroids and IL-6 blockers y/n | Increase 0.411 | Admission Hb, CRP and MV duration | P<0.001 |

Supplementary Table 5. Impact of steroids adjusted for significant covariates on number of red blood cell transfusions

| Co-variate | Nr of RBC transfusions | Adjusted for | P value |
| --- | --- | --- | --- |
| Per 1 hour MV | Increase 0.006 units | Steroids | P=0.006 |
| Steroids y/n | No change | MV duration | P= 0.156 |

Supplementary Table 6. Impact of combination treatment adjusted for significant covariates on number of red blood cell transfusions

| Co-variate | Nr of RBC transfusions | Adjusted for | P value |
| --- | --- | --- | --- |
| Per 1 hour MV | Increase 0.006 units | Treatments | P<0.039 |
| Steroids and IL-6 blockers y/n | Decrease 1.924 units | MV duration | P= 0.156 |

Supplementary Figure 1.
